# Precrastination and Time Perspective: Evidence from Intertemporal Decision-Making

**DOI:** 10.3390/bs13080631

**Published:** 2023-07-28

**Authors:** Boyang Ma, Yong Zhang

**Affiliations:** School of Education and Psychology, Southwest Minzu University, Chengdu 610225, China; maboyang@stu.swun.edu.cn

**Keywords:** precrastination, time perspective, proactive personality, intertemporal decision-making

## Abstract

Although procrastination has been extensively studied, precrastination remains an unsolved puzzle. Precrastination is the tendency to start tasks as soon as possible, even at the cost of extra effort. Using the near bucket paradigm with 81 undergraduate students, this study examined the relationship between precrastination and time perspective, proactive personality, and subjects’ differential performance in intertemporal decision-making. The results confirmed the cognitive-load-reduction (CLEAR) hypothesis. Precrastination was found to be positively predicted by the future time dimension of time perspective and negatively predicted by proactive personality. In addition, there is a significant positive correlation between precrastination and delay discounting of intertemporal decision-making, which exists only for the loss situation.

## 1. Introduction

Procrastination is a common tendency observed in the habits of behavior and coping strategies of individuals. Previous research has shown that chronic procrastination is present in as many as 15–20 percent of adults across different cultures [1]. However, in a study investigating the biomechanical balance between distance and weight, Rosenbaum et al. stumbled upon a phenomenon that is the exact opposite of procrastination, which they call precrastination. Precrastination is the tendency to complete a task quickly, but only in order to complete it sooner rather than later, even if it triggers unreasonable choices and consumes more energy or resources [2]. Common examples include arriving at the airport six hours early with nothing else to do or responding to an email immediately rather than thinking about it first. Precrastination usually occurs when people are assigned a task and they complete it immediately without thinking about a better solution, wasting more time, money and mental effort. Current mainstream theory suggests that starting or completing a task earlier allows an individual to offload cognitive load more quickly [2,3], to clear their mind [4], and to reduce stress on prospective or working memory [5,6], thereby leaving more cognitive resources available for other tasks. On the other hand, precrastination may be an automatic tendency to respond [7] or to respond automatically to stimuli or demands [8], such as the mere urgency effect that has recently been discussed [9]. In addition, other researchers have used the aiming hypothesis as an explanation for precrastination [10]. Experimental evidence [2,7] suggests that the core of precrastination is not task completion, but rather the acceleration of subgoal initiation.

Several studies have found very similar rates of precrastination [2,11], suggesting that there are consistent individual differences in precrastination. Precrastination is positively correlated with both conscientiousness and agreeableness [11], but not with impulsiveness or self-regulation [12], which seems to contradict Fournier’s predictions [7]. Raghunath’s [13] experiment found that precrastination was not associated with procrastination and was not correlated with working memory span but was associated with decreased enjoyment and engagement in thinking. Gehrig et al. found that precrastination is more than just the opposite of procrastination; precrastination shows a significant increase in explained variance of personality traits over procrastination alone [14].

In Rosenbaum’s classic bucket experiment [2], the majority of participants demonstrated a tendency to precrastinate by carrying the bucket closer to the end, despite the increased physical effort involved. However, there were still some participants who did not choose to precrastinate. Why did these participants make different choices? Whether it was deciding to precrastinate or delay carrying the bucket, there appears to be an underlying reflection of participants’ attitudes towards time. Could it be possible that the variations in their choices were influenced by their different perceptions of time? Research on procrastination has indicated that one’s time perspective can have an impact on procrastination behavior [15]. Zimbardo and Boyd suggest that people typically divide their coherent lives into different temporal categories, including past, present, and future, and that they hold relatively stable attitudes towards different temporal categories [16]. These attitudes may be positive or negative, and such temporal divisions and attitudes give order, coherence, and meaning to our lives. Time perspective is one of the most important factors influencing how people think, feel, and act, and people tend to develop and overuse a particular time perspective that reflects a person’s attitudes, beliefs, and values about time. Precrastination reflects a person’s response to time [10] and may be potentially related to a person’s time perspective. Furthermore, precrastination may also be related to the work environment and task pressure [10]. An individual with a proactive personality is usually less constrained by the environment and instead chooses, or even creates, an environment to achieve high work performance. Therefore, it is worth exploring the relationship between precrastination and time perception and proactive personality.

If precrastination is a fundamental phenomenon, then it could also occur in non-human animals. Wasserman’s experiments have demonstrated that precrastination can occur in pigeons [17], despite the lack of any differential reward for doing so, the pigeons will still choose to precrastinate. This suggests that precrastination is not unique to humans, but is common across species. Precrastination may involve several different levels of mental processes, including simple automatic responses, immediate information processing or working memory, and decision-making or judgement. Previous research has primarily focused on the first two levels of analysis. However, in our study, we aimed to delve deeper into the relationship between thinking and precrastination, building upon existing findings [18]. Fournier and colleagues discovered that individuals strategically employ precrastination, adjusting their choices based on factors such as distance, cognitive load, and task completion. This suggests that thinking plays a distinctive role in human precrastination [7]. Furthermore, intertemporal decision-making reflects individuals’ thinking preferences under varying situational conditions. Previous studies have demonstrated that participants’ perceived time delay significantly influences the outcomes of their intertemporal decision-making, and a positive correlation exists between time perspective and intertemporal decision-making [19]. Hence, it is reasonable to hypothesize that individuals with different tendencies towards precrastination may exhibit varying degrees of delay discounting in their intertemporal decision-making. Exploring this further will provide valuable insights into the relationship between precrastination and time perspective.

In summary, precrastination encompasses the participant’s decision-making process regarding immediate action versus delay, while also considering the understanding of present and future time. Consequently, individuals with different time perspectives may exhibit varying rates of precrastination. Based on this observation, we propose:

**Hypothesis 1.**  *Precrastination will have a positive correlation with present-hedonistic tendencies and a negative correlation with future time orientation. Individuals who prioritize immediate pleasure (present-hedonistic) are more likely to engage in precrastination, while those with a stronger focus on the future are less likely to choose precrastination*.

Proactive personality refers to an individual’s inherent inclination to take proactive measures and explore new ways to exert influence on the external environment, disregarding situational constraints. We believe that individuals with a proactive personality possess the capacity to adapt their behavior effectively in dynamic environments. Rather than engaging in spontaneous precrastination, individuals with a proactive personality tend to thoroughly explore all possible options. Therefore, we propose:

**Hypothesis 2**  . *Proactive personality positively predicts precrastination*.

Intertemporal decision-making aligns with precrastination as it involves individuals making choices between two time points—present and future. However, the fundamental nature of precrastination centers around loss rather than gain, leading to different psychological effects between the two. Based on this observation, we design two scenarios within the intertemporal decision task and formulate:

**Hypothesis 3**  . *Individuals prone to precrastination display distinct performance in these two scenarios of intertemporal decision-making, exhibiting a higher delayed discounting rate specifically in the loss scenario*.

## 2. Materials and Methods

### 2.1. Participants

This study was approved by the Ethics Committee of Southwest Minzu University. A group of 84 undergraduates were initially recruited to participate in the experiment. Three participants were excluded because they failed to report their calculations in a timely manner while performing a carrying task with cognitive load. Finally, 81 valid participants were retained (63 were women; age range was 18–23 years, M = 20.12 years). Using the dominant hand questionnaire modified by Oldfield [20] and Brown et al. [21], 79 participants were identified as right-handed (score ≥ 0.5), 2 participants as left-handed (score ≤ −0.5), and 1 participant with different handedness (−0.5 < score < 0.5). None of the participants had cognitive impairment, color blindness, or color amblyopia. All participants had normal vision or corrected-to-normal vision, and they were paid 16 CNY at the end of the experiment.

### 2.2. Stimuli and Apparatus

#### 2.2.1. Task of the near Bucket

**Figure 1:**
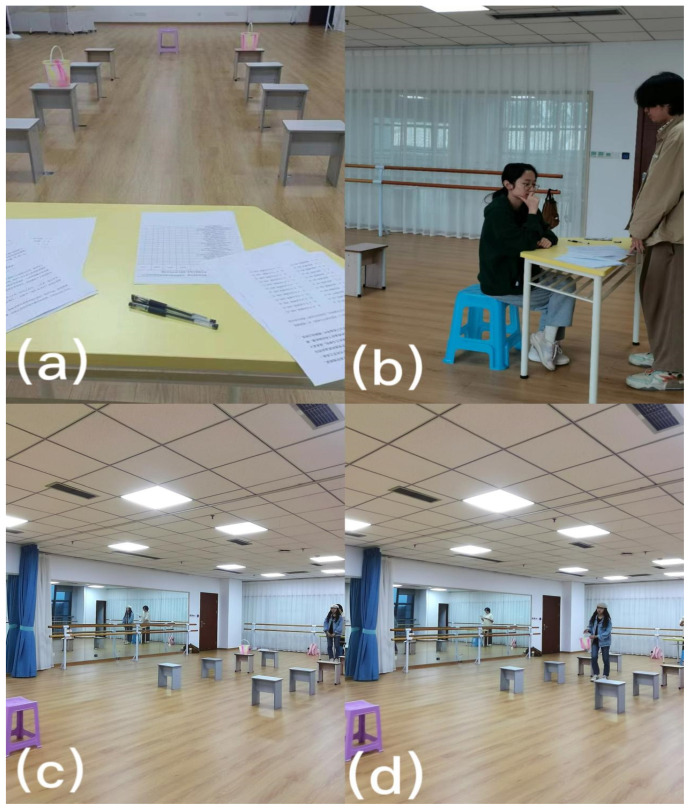
Experimental scenario. After filling out the questionnaire, the participant began the carrying task. Using the amounts in feet as the coordinates, the participant in this example chose the bucket on the right, which was approximately 8 feet away from the starting point at position (4, 8). The 4 and the 8 represent the two buckets placed on the iron stool, approximately 4 feet to the left and 8 feet to the right of the starting point. In this figure, the following sequence of events is depicted: (**a**) The experimental scene was set up before the participant arrived; (**b**) The participant arrived and completed the questionnaire, which included measurements of time perspective, proactive personality, and the intertemporal choice task; (**c**) After completing the questionnaire, the participant stood up, turned around, and prepared to begin the near bucket task; (**d**) The participant walked through the corridor, selected one of the buckets, and carried it to the endpoint.

The experimental scenario is shown in Figure 1. The experimental apparatus was as follows: a yellow wooden table (0.77 m high, 1.18 m long, 0.615 m wide); eight iron stools (0.4 m high, 0.363 m long, 0.24 m wide), four on each side, 1.2 m (approximately 4 feet), 2.4 m (approximately 8 feet), 3.6 m (approximately 12 feet), and 4.8 m (approximately 16 feet) from the table, respectively, marked on the floor with black tape; two translucent plastic buckets (0.216 m high, 0.244 m diameter), each containing an orange ping-pong ball to allow subjects to more clearly identify the water level; two plastic stools (0.27 m long, 0.27 m wide, 0.44 m high), the blue stool located in front of the wooden table for participants to fill in the questionnaire, and the purple stool located at the center of the end of the corridor (7.2 m from the starting point) for the bucket. Participants start at the yellow table in Figure 1a and end at the purple plastic stool in Figure 1c. Assuming a straight line between the start and end points, the eight iron stools are evenly located 0.805 m to the left and right of the hypothetical straight line.

#### 2.2.2. Measurement of Time Perspective

The Zimbardo Time Perspective Inventory (ZTPI) [16] was chosen to divide time perspective into five dimensions based on the individual’s experience of the past, present, and future. The five dimensions can be specifically divided into: past negative, past positive, present predestined, present-hedonistic, and future time. Both precrastination and procrastination involve participants’ cognition of present and future time. Therefore, two dimensions of ZTPI, present-hedonistic and future time, were chosen to be measured. The present-hedonistic is the degree of tendency to focus on pleasure, adventure, sensory stimulation, and timely satisfaction. This dimension consists of 15 questions (e.g., I think it’s more important to enjoy what I’m doing now than to get things done on time, I think it’s important to seek excitement in my life) and those who scored high on this dimension proved to be more inclined towards immediate gratification and less likely to think about the future. Future time is a factor that points to the future and creates motivation for the future. This dimension consisted of 13 questions (e.g., To achieve my dreams, I will set goals and consider specific steps to achieve them, Being late for appointments annoys me), including three reverse-scored questions, and those who scored higher on this dimension were more likely to think about the future and set clear goals. The questionnaire used the Likert five-point scoring method with a total of 28 items. Higher scores in each dimension indicate a stronger temporal orientation in the corresponding dimension.

#### 2.2.3. Measurement of Proactive Personality

Proactive personality refers to an individual’s tendency to act on their own initiative to change their external environment, without being constrained by situational resistance. The Proactive Personality Scale (PPS), revised by Zhang, Z. et al. [22] from Bateman and Grant [23], was chosen, which uses a five-point scoring method with a total of 17 questions (e.g., If I believe in something, I will do it regardless of the likelihood of success or failure, Even if others are against it, I will stick to my idea). Higher scores on the scale indicate higher levels of proactive personality in the subjects. The internal consistency coefficient of the scale is 0.824. The results of the validation factor analysis showed that all fitted indicators for the Proactive Personality Scale were within acceptable limits, indicating that the scale has good construct validity.

#### 2.2.4. Task of Intertemporal Choice

Intertemporal Choice involves the costs and benefits of making decisions at different points in time. The discount rate for delayed outcomes varies depending on whether the outcome is a loss or a gain. Similarly, precrastination involves a trade-off between people’s choices of near and far buckets (paying extra physical effort or bearing a cognitive burden). Based on this, we adapted the Intertemporal Choice Questionnaire by Chen, H. and He, G. [24] and developed two different versions of the questionnaire, one for the gain (reward) and another for the loss (punishment). The gain (loss) scenario is that your company makes a profit (loss) and pays a bonus (subject to a fine), but the amount gained (lost) will vary depending on when you get the bonus (pay the fine). The available options are as follows:

1. A. Receive 50 CNY (lose 950 CNY) now B. Receive (lose) 1000 CNY after 6 months.
2. A. Receive 100 CNY (lose 900 CNY) now B. Receive (lose) 1000 CNY after 6 months.

……

19. A. Receive 950 CNY (lose 50 CNY) now B. Receive (lose) 1000 CNY after 6 months.

For the results obtained, the delay discount rate of the participants was calculated using the formula V = A/1 + KD, and k was normally transformed to obtain lnk, where V is the value, the subjective value point in the individual’s mind; A is the amount of option, the reward price after delay; D is the delay, the time of delay, which can be measured in seconds, minutes, hours, days, months, and years; K is the discount rate, the higher the value of K, the stronger the preference for immediate rewards and the lower the degree of waiting for temptation, and the lower the value of K, the stronger the preference for delayed rewards.

### 2.3. Design and Procedure

A 2 (cognitive load task: yes vs. no) × 2 (intertemporal choice: gain vs. loss) within-subjects design was used. This study examined Rosenbaum’s near bucket experiment [2] and Blinch’s block-grabbing design [8] and combined them. Specifically, the experimenter placed buckets containing water on the left and right sides of the hallway and used the distance of the buckets from the starting point as coordinates. Using the number of feet from the starting point, the buckets were positioned in ten scenarios—(4,8); (8,4); (4,12); (12,4); (4,16) and (16,4); (8,12); (12,8); (8,16); (16,8). Participants were asked to choose the left or right bucket in their preferred manner to complete the task and then return to the starting point to wait for the next trial. The first five of the ten scenarios had no cognitive load, i.e., the participant only had to complete the task and bring the bucket to the end of the line, while the second five had a cognitive load, which was controlled by giving the participants a fixed number and asking the participant to keep doing the minus three calculation for that number. During this process, the experimenter recorded whether the participant took the bucket early and the results of the calculation.

The experiment was conducted in a closed room, with the experimenter and two translucent buckets containing 1.555 L of water (each with an orange ping-pong ball inside to help participants judge the level of the water) hidden in advance, and eight stools on either side to hold the buckets to prevent walk-in participants from seeing the placement of the experiment in advance and getting a better idea of the expected location or guessing the purpose of the experiment.

While the participant was filling out the questionnaire, the experimenter placed the iron stool and bucket on the pre-applied black tape (see Section 2.2.1) and in the appropriate locations. After completing the questionnaire, the participant could start the carrying task without cognitive load. The participant was asked to take either bucket, place it on the final stool, and return to the starting point facing the experimenter, who had quickly changed the starting position of the bucket. It is important to note that when describing the rules of the task, the experimenter did not ask participants which hand they could use to pick up or put down the bucket, nor did he deliberately limit the time to complete the task, but only emphasized that participant could complete the task in a way that was comfortable for them.

After understanding the rules, the participant reported “ready” to the experimenter. When the experimenter responded “go”, the participant could turn around and was given three seconds to observe the field before beginning the carrying task. The single trial was repeated five times. The experimenter recorded the participant’s choice of the near bucket as precrastination and the choice of the far bucket as non-precrastination.

After completing the carrying task without cognitive load, the participant started the carrying task with cognitive load. Participant was given one number at a time and asked to calculate the number in their head by subtracting three while carrying and to report the results aloud at the end of the session, which kept participants’ cognitive resources in a constant state of processing. The numbers were generated by a random number generator: 197, 256, 398, 985, 635. The experimenter did not emphasize the need for a minimum number of subtractions or the need for correctness, but the other settings were the same as before. The experimenter recorded whether the subject chose to proceed or not, and the results of the calculation of the numbers. The results of the calculations were divided into three categories: correct, incorrect, and calculated only once.

At the end of the experiment, participants were asked about their reasons for their choice with and without cognitive load, and their preferred hand was surveyed. Each participant received a reward of 16 CNY.

## 3. Results

### 3.1. Precrastination’s Occurrence Rate

In the ten carrying tasks conducted, a total of 79 participants chose to precrastinate at least once, whereas three participants consistently refrained from precrastinating. Thus, the overall precrastination rate observed in this study was 96%. Participants were subsequently asked to provide explanations for their choices across varying cognitive load conditions, and their responses are summarized in Table 1.

Without cognitive load, 19 participants always chose the bucket closer to the finishing point, and 62 participants chose the closer bucket at least once, i.e., precrastination. The total rate of precrastination was 76.5%, with 33.3% of participants reporting wanting to take the bucket closest to themselves and 23.5% reporting wanting to take the bucket closest to the finishing point. However, with addition of cognitive load, only seven participants always chose the bucket closest to the finishing point, and the remaining 74 participants chose the closer bucket at least once, resulting in an overall rate of precrastination of 91.4%. A paired sample T-test was conducted on the rate of precrastination with and without cognitive load, and the results showed a significant increase in the rate of precrastination among participants with cognitive load (t = −5.983, *p* < 0.01, Cohen’s d = 0.78). This implied a strong preference towards choosing the bucket closest to themselves while adding cognitive load. Out of the seven participants who always chose the bucket closest to the finishing point, only one participant completed the cognitive task correctly, while the other six had made calculation errors or only calculated once. In fact, this was common among participants who chose both precrastination and non-precrastination—those who changed their choices suddenly were more likely to make calculation errors or only calculate once. When participants were asked about the reasons for their choices, almost half said they were overwhelmed and just wanted to relieve their mental load.

### 3.2. Correlation Analysis between Precrastination and Future Time and Proactive Personality

The correlation analysis between time perspective and the rate of precrastination revealed that future time orientation was significantly positively correlated with the overall rate of precrastination (r = 0.295, *p* = 0.007). And there was a significant positive correlation between future time orientation and the rate of precrastination in the group without cognitive load (r = 0.276, *p* = 0.016). However, there was no significant correlation between ‘present-hedonistic’ and the three types of precrastination rates. These results suggested that individuals who were more focused on future time orientation in terms of time insights were more likely to be involved in precrastination.

Through Pearson correlation analysis between the rate of precrastination and proactive personality, it was found that precrastination was negatively correlated with proactive personality when cognitive load was present (r = −0.226, *p* = 0.042), but there was no significant correlation between precrastination and proactive personality when there was no cognitive load and in the overall rate of precrastination. There was no significant correlation between future time, present-hedonistic, and cognitive load through a mixed repeated measurement analysis.

Through regression analysis between “present-hedonistic”, future time orientation, proactive personality, and rate of precrastination under different cognitive loads, it was found that the regression coefficient between future time orientation and total rate of precrastination was significant (F = 7. 56, *p* = 0.007, R^2^ = 0.087), and the regression coefficient between future time orientation and precrastination rate under no cognitive load was significant (F = 6.04, *p* = 0.016, R^2^ = 0.071). This suggested that future time orientation can positively predict the occurrence of precrastination. Furthermore, the regression coefficient between proactive personality and the rate of precrastination under cognitive load was significant (F = 4.259, *p* = 0.042, R^2^ = 0.051), but no other variable had a significant regression coefficient (*p* > 0.05).

### 3.3. Precrastination in Intertemporal Decision-Making Tasks

In this study, participant’s subjective value point V was obtained through their choice of questionnaires, and then their discount rate was calculated using the formula V = A/1 + KD, fitting a hyperbolic curve model through lnk. By analyzing the correlation between the precrastination rate under cognitive load and the integral difference in the gain or loss situation, it was found that the total precrastination rate was positively correlated with delay discounting in the loss situation of intertemporal choice (r = 0.323, *p* = 0.003). The precrastination rate of the cognitive load group was positively correlated with delay discounting in the loss situation of intertemporal choice (r = 0.320, *p* = 0.004). However, there was no significant correlation between participants’ precrastination rate and delay discounting in the gain situation of intertemporal choice.

## 4. Discussion

In this study, we aimed to replicate and expand upon previous precrastination research. Specifically, we created scenarios that involved both task load and varying bucket position coordinates. We recorded participants’ precrastination rates as well as their calculation accuracy, and further investigated the factors influencing their choices between tasks with and without cognitive load. It is essential to discuss the above findings in detail.

### 4.1. The CLEAR Hypothesis Was Confirmed

This study found that most subjects showed at least one precrastination in the near bucket task. Subjects’ precrastination rates increased when a working memory task was added. Firstly, this suggests that participants’ precrastination rates in the present study are very close to those found in previous studies [2,6,7]. This is the first time that precrastination has been investigated in East Asian subjects. These results suggest that precrastination is universal across cultures. Using a modified classical bucket paradigm, this study found results consistent with previous studies. This indirectly confirms the reliability of the experimental manipulation. Second, those in the cognitive load group had significantly higher precrastination rates than those in the non-cognitive load group. When there are multiple tasks to be completed, working memory load influences the preferential selection of the goal. The subjects are more likely to precrastinate. People are reluctant to hold the need to do one thing in their working memory for long periods of time. They tend to try to get one thing or one of the items off their mental to-do list [2,4].

### 4.2. Precrastination Can Be Predicted by Future Time Perspective and Proactive Personality

The hypothesis that precrastination is related to time perception was confirmed in this study. Specifically, there was a significant positive correlation between precrastination and future time perspective. Future time perspective is usually characterized by a person’s ability to plan and achieve future goals, reflecting an overall future orientation. The results suggest that individuals with a high future time perspective are more likely to experience precrastination. This suggests that precrastination reflects participants’ sense of time urgency and the immediate need for task goals. However, this result is surprising as it contradicts the initial expectation that participants who exhibit present-hedonistic tendencies would be more likely to choose precrastination. One possible explanation for this discrepancy is that participants with high future time orientation scores are motivated to expedite the completion of the current task in order to allocate more cognitive re-sources towards long-term plans or goals. Consequently, they opt for precrastination. Some researchers have claimed that precrastination is a result of participants’ desire to start the task immediately, i.e., to accelerate the start of the sub-goal [5]. This finding ap-pears to support this viewpoint. On the other hand, having a present-hedonistic tendency allows participants to concentrate on the current task and provides an opportunity to break free from automatic respond behavior.

Furthermore, proactive personality tendencies can also serve as predictors of precrastination behavior. Participants with higher scores in proactive personality were found to be less prone to precrastinate. This finding aligns with the predictions put forth by the automatic response hypothesis and the mere urgency effect [5,7,9]. However, no significant relationship was observed between proactive personality and precrastination rates in situations without cognitive load. One possible explanation is that this connection may not be apparent or active when cognitive load is low. On the other hand, under high cognitive load conditions, where individuals have limited cognitive resources available, they tend to hastily deal with changes and show psychological advantages. Proactive personality traits enable individuals to adapt and interact proactively with their environment, resulting in a moderating effect in such circumstances. The presence of proactive personality assists participants in adopting a global perspective of the entire task, allowing them to transcend automatic responses and reduce the likelihood of precrastination.

### 4.3. Precrastination Is Related to Delayed Discounting in the Loss Task for Intertemporal Decision-Making

The results confirm the hypothesis of this study that precrastination is related to intertemporal decision-making. This is not surprising, as previous research has shown a positive relationship between time perspective and delay discounting. In intertemporal decision-making, participants gain time discounts for immediate gratification and are unwilling to delay gratification; they lose time discounts to avoid immediate discomfort in the immediate future and would rather suffer higher costs in the future. This is logically consistent with end-state comfort theory [10]. Interestingly, however, in the present study, precrastination was not correlated with participants’ delay discounting in the gain task of intertemporal decision-making, but was significantly positively correlated with participants’ delay discounting in the loss task of intertemporal decision-making. This implies, firstly, that one of the reasons for precrastination is fear of future loss. It was the fear of possible failure and losses in the subsequent task that led participants to precrastinate. Secondly, the results of this study suggest a biological tendency to respond asymmetrically, i.e., a far greater aversion to loss than convergence towards gain. This is almost perfectly consistent with the logic of Tversky and Kahneman’s framing effect. Indeed, some researchers have found that subjects also show asymmetric responses in both the gain and loss conditions of inductive reasoning [25]. Others have also found asymmetry in subjects’ delay discounting and corresponding neural activity in the gain and loss conditions of intertemporal decision-making [26,27]. This strongly confirms that precrastination is not only an automatic or an immediate response, but may involve more complex cognitive processes such as reasoning and decision-making.

### 4.4. Shortcomings and Considerations

This study also encountered some unexplained flaws or findings that require further investigation. For example, in the calculation task, participants were explicitly instructed to keep subtracting three while carrying, but there was no specific emphasis on the need to perform multiple calculations or ensure accuracy. As a result, it was observed that some participants only performed a single calculation, while others made errors in their calculations. However, this outcome was anticipated given our experimental design, as the cognitive load control condition for this calculation task differs from previous studies [7]. The process of constantly subtracting three for a given number is dynamic and requires increasing cognitive processing steps as the number of calculations rises, thus increasing the likelihood of errors. We suggest that participants who are more prone to precrastination may have a tendency to reduce task load and achieve mental load reduction by minimizing the number of calculations, such as taking the bucket only once.

After the experiment, we further confirmed that participants who only performed the minus three task once had a clear understanding of the task rules and the rationale behind performing the task in this manner. All participants reported that they comprehended the rules of the task, with the majority explaining that they found it challenging to carry the bucket while continuously subtracting three or that they were prone to making mistakes if they continued counting. Consequently, they chose to prioritize speed over extensive thinking. Interestingly, these findings align with the results of the initial precrastination experiment [2], which indicated that individuals tend to seek mental comfort by engaging in additional physical effort. This reinforces the hypothesis that participants seek to clear their minds or maintain a relaxed state, opting for reduced cognitive processing to achieve this desired mental state.

In the experimental procedure of this study, the non-cognitive load and cognitive load tasks were administered in a predetermined sequence. Specifically, participants first completed the non-cognitive load task, followed by the cognitive load task. This ordering was based on findings from the pre-experiment phase, which revealed that if the cognitive load task was performed first, participants were more likely to develop a practice effect that influenced their subsequent responses in the following five trials. To address this issue, the current study ensured that participants had sufficient cognitive resources available for processing the task during the initial five trials. However, it is important to note that this approach may potentially result in a reverse practice effect. As this effect being relatively weaker compared to the alternative, the study chose to use the existing procedure. Nevertheless, it is critical to acknowledge this limitation and tackle it in future research.

## 5. Conclusions

In this study, precrastination was found to be positively predicted by the future time dimension of time perspective and negatively predicted by proactive personality. In addition, there is a significant positive correlation between precrastination and delay discounting in intertemporal decision-making, which exists only for the loss situation.

## Figures and Tables

**Table 1:** Reasons reported by participants (N = 81).

Cognitive Load	Reason	Number of People	Rate
Without cognitive load	It was more convenient to take the closer bucket.	27	33.3%
	Taking the bucket that was closer to the end point saved energy.	19	23.5%
	Considering that the distance between choosing a single bucket and two buckets was related.	11	13.6%
	Considering it as a subconscious behavior, took the bucket without thinking.	9	11.1%
	Took the bucket based on feeling, without thinking.	4	4.93%
	Believe the task was simple and chose the one that seemed easier to complete.	4	4.93%
	Wanted to try to move to both sides and change things up.	4	4.93%
	Carried water bucket to the opposite side of cellphone to avoid imbalance caused by weight difference.	1	1.23%
	Lucky number is two, considering the left as one and the right as two, so took the bucket on the right each time.	1	1.23%
	Preferred the left hand and used the right hand only for picking up a bucket (The participant exhibited mixed handedness).	1	1.23%
With cognitive load	Felt too busy in my head, so took what is closer first.	38	46.9%
	Subconsciously chose the closer option during the calculation process.	15	18.5%
	It was more convenient to pick up the bucket earlier for easier calculation.	8	9.87%
	Initially picked near bucket for calculations and eventually switched to far bucket for better results.	6	7.41%
	Believed that the task was not difficult and took the bucket that was closer to the end point.	6	7.41%
	Wanted to try both buckets, but they preferred the closer one.	3	3.70%
	Believed that calculating first would be better to obtain the bucket.	2	2.47%
	Carrying a bucket from one side only improved mechanical movement for better calculation.	2	2.47%

## Data Availability

The data presented in this study are available on request from the corresponding author.
